# Location of the Central Retinal Vessel Trunk in the Laminar and Prelaminar Tissue of Healthy and Glaucomatous Eyes

**DOI:** 10.1038/s41598-017-10042-5

**Published:** 2017-08-30

**Authors:** Bo Wang, Katie A. Lucy, Joel S. Schuman, Hiroshi Ishikawa, Richard A. Bilonick, Ian A. Sigal, Larry Kagemann, Chen Lu, James G. Fujimoto, Gadi Wollstein

**Affiliations:** 1 UPMC Eye Center, Eye and Ear Institute, Ophthalmology and Visual Science Research Center, Department of Ophthalmology, University of Pittsburgh School of Medicine, Pittsburgh, PA USA; 2 Department of Bioengineering, Swanson School of Engineering, University of Pittsburgh, Pittsburgh, PA USA; 3 NYU Langone Eye Center, New York University School of Medicine, New York, NY USA; 4 Department of Biostatistics, Graduate School of Public Health, University of Pittsburgh, Pittsburgh, PA USA; 5 Center for Devices and Radiological Health, Food and Drug Administration, Silver Spring, MD USA; 6 Department of Electrical Engineering and Computer Science, Massachusetts Institute of Technology, Cambridge, MA USA

## Abstract

Glaucoma is a leading cause of blindness that leads to characteristic changes in the optic nerve head (ONH) region, such as nasalization of vessels. It is unknown whether the spatial location of this vessel shift inside the ONH occurs within the lamina cribrosa (LC) or the prelaminar tissue. The purpose of this study was to compare the location of the central retinal vessel trunk (CRVT) in the LC and prelaminar tissue in living healthy and glaucomatous eyes. We acquired 3-dimensional ONH scans from 119 eyes (40 healthy, 29 glaucoma suspect, and 50 glaucoma) using optical coherence tomography (OCT). The CRVT location was manually delineated in separate projection images of the LC and prelamina. We found that the CRVT in glaucoma suspect and glaucomatous eyes was located significantly more nasally compared to healthy eyes at the level of the prelamina. There was no detectable difference found in the location of the CRVT at the level of the LC between diagnostic groups. While the nasal location of the CRVT in the prelamina has been associated with glaucomatous axonal death, our results suggest that the CRVT in the LC is anchored in the tissue with minimal variation in glaucomatous eyes.

## Introduction

Glaucoma, the second leading cause of blindness worldwide, is an optic neuropathy resulting in vision loss due to the death of retinal ganglion cell axons^1^ Axonal death results in characteristic changes that occur at the level of the optic nerve and retina^2, 3^. These changes include a shift of the central retinal vessels, often nasally, which has been observed clinically and experimentally in glaucomatous eyes^4–6^ and therefore can be used to characterize abnormal glaucomatous discs.

The location of the central retinal vessel trunk (CRVT) is a topic of clinical significance, as its location has been associated with susceptibility to glaucomatous damage and peripapillary atrophy^5, 7^. Regions of the neuroretinal rim that are close to the CRVT appear to be less affected by glaucomatous damage until late into the disease. The position of the CRVT has also been associated with vision loss as measured with visual field testing, especially in advanced glaucoma^8, 9^. Central vision loss in glaucoma was seen more often in eyes with nasally positioned CRVTs, while eyes with preserved central visual field islands tended to have a more temporal CRVTs. As such, it is important to better characterize the location of the CRVT in healthy and glaucomatous eyes in order to improve our understanding of the glaucomatous process. The location within the optic nerve head (ONH) where vessel shifts occur in glaucoma is currently unknown. Shifts could occur at the level of the LC, at the level of prelaminar tissue, or both.

The composition of the LC and prelamina of the ONH are different, potentially affecting the location of vessel displacement. The LC is composed primarily of connective tissue made of densely packed collagenous fibers, which is anchored to the scleral tissue that surrounds it. The prelaminar tissue is composed primarily of retinal ganglion cell axons and a loose arrangement of supportive glial cells. The prelaminar layer thins as glaucoma progresses, reflecting the damage and loss of retinal ganglion cells. It is currently unknown whether damage to the LC in glaucoma results in differences in the CRVT location at the level of the LC. The purpose of this study was to test the hypothesis that the retinal vessel shift seen in glaucoma occurs at the level of the prelaminar tissue.

## Methods

### Study Subjects

The study was conducted in accordance with the Declaration of Helsinki and the Healthy Insurance Portability and Accountability Act. The institutional review board of the University of Pittsburgh approved the study, and all subjects gave written informed consent prior to participation.

Eighty four subjects, including 25 healthy (35 eyes), 19 glaucoma suspect (27 eyes), and 40 subjects with glaucoma (49 eyes) were included in the study. All subjects underwent a comprehensive clinical examination, including a review of medical history, measurement of best corrected visual acuity, refraction, slit lamp biomicroscopy exam, intraocular pressure measurement, visual field (VF) testing, and scanning with optical coherence tomography (OCT). To be included in this study subjects needed a visual acuity of 20/60 or better and a spherical equivalent refractive error between −6.00 and + 6.00 diopters. Subjects were excluded if they had a history of diabetes, any conditions affecting VF and retinal thickness other than glaucoma, a history of ocular trauma, or any surgeries other than uncomplicated glaucoma interventions or cataract extraction. Additionally, subjects were excluded for the use of any medication known to affect the retina.

Healthy subjects were classified based on having the appearance of a healthy optic nerve, as determined during clinical examination, a full visual field, and no other ocular pathologies. Glaucoma suspects were classified as those with normal VF results in the presence of elevated intraocular pressure (>21 mmHg), clinically suspicious ONH appearance (large cupping, neuroretinal rim notch, asymmetry of cupping between eyes), retinal nerve fiber layer (RNFL) defect, or if the eye was the contralateral eye of a unilateral primary open angle glaucoma subject. Glaucomatous eyes were classified as eyes with characteristic glaucomatous damage (as detailed above for the glaucoma suspects) with corresponding and reproducible VF loss. If both eyes were qualified, they were included in the appropriate category.

### VF testing

All subjects had reliable Swedish interactive thresholding algorithm (SITA) standard 24-2 perimetry testing (Humphrey Field Analyzer, Zeiss, Dublin, CA). Qualified VF examinations had less than 33% fixation losses, false positive, and false negative responses. Glaucomatous VFs were defined as those with at least one of the following confirmed on at least two consecutive visits: glaucoma hemifield test (GHT) outside normal limits or pattern standard deviation (PSD) probability outside the highest 9% of the healthy population.

### OCT Image Acquisition

A swept-source (SS)-OCT scan centered on the ONH was acquired from all subjects. The SS-OCT featured a 1050 nm central wavelength with a 100 kHz scan rate^10^. The device had an axial resolution of 5 μm and lateral resolution of 20 μm in tissue. The scanning volume was 3.5 × 3.5 × 3.64 mm (400 × 400 × 896 samplings), with the focus adjusted to the level of the LC. Two orthogonal scan volumes were acquired sequentially and automatically registered to reduce motion artifacts^11^. Subjects with poor LC visibility were excluded from the analysis, as it was not possible to determine the location of the vessel trunk at the level of the LC in these images.

### Delineation of Central Retinal Vessel Trunk

Prior to delineation, the scans were made isotropic by upsampling in the X and Y direction and rotating so that the Bruch membrane opening (BMO) was flat. The manual en face delineation of the CRVT and the BMO was performed by an experienced observer that was masked to subject identity and all clinical information, and was done by using the FIJI^12^ software, based on the National Institute of Health’s ImageJ program^13^.

**Figure 1:**
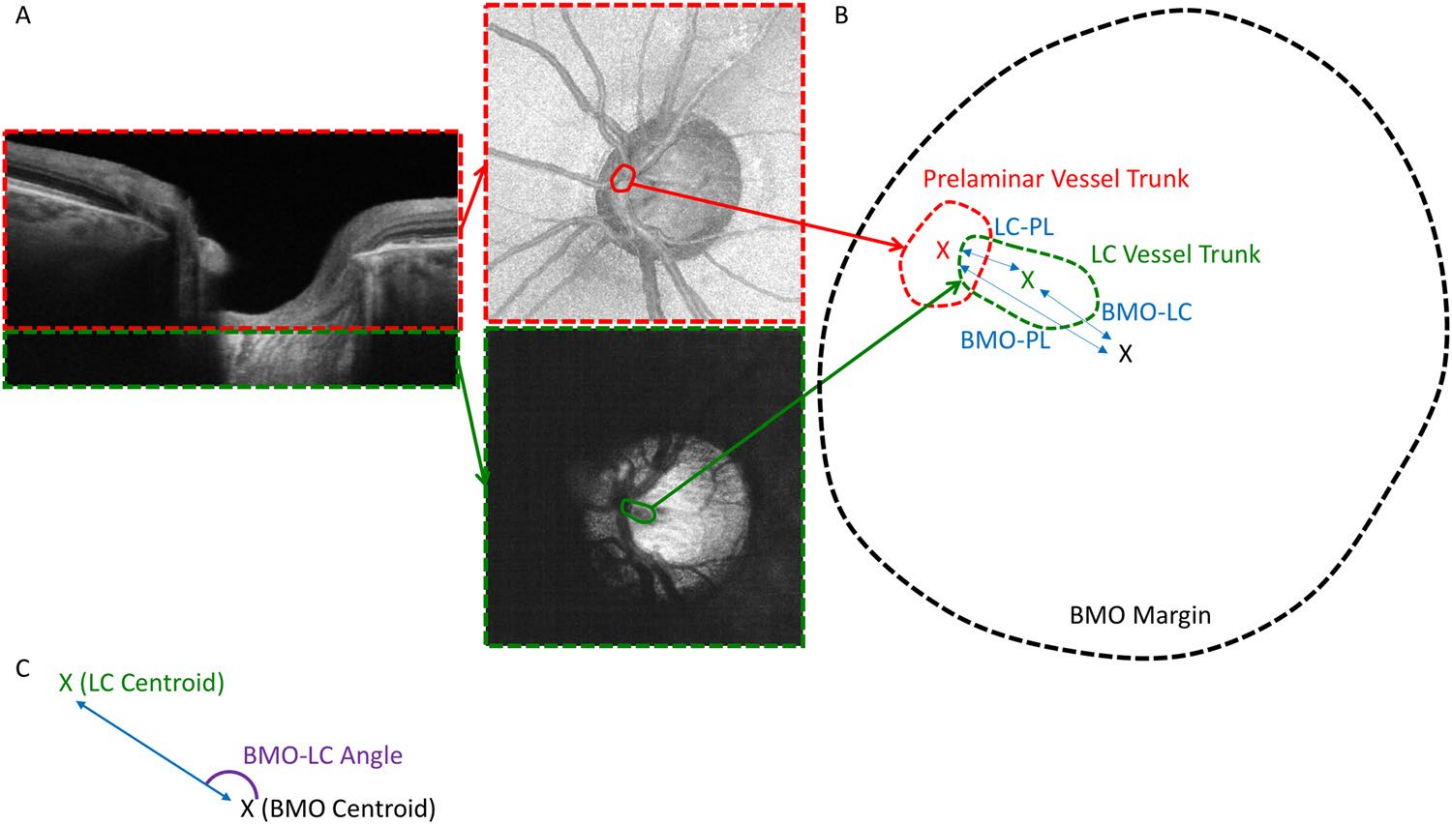
(**A**) Cross-sectional scan divided at the posterior boundary of the prelaminar tissue (red dashed line) and anterior lamina cribrosa (LC) surface (green dashed line), from which two projection images were formed. Delineation of the central retinal vessel trunk (CRVT) is marked in each location (red and green circles). (**B**) Illustration of the three relationships measured: 1) the center of the Bruch membrane opening (BMO) and the LC CRVT (BMO-LC), 2) the BMO center and the prelaminar CRVT (BMO-PL), and 3) the LC CRVT and the prelaminar CRVT (LC-PL). X denotes the centroid of the region (red – prelaminar CRVT centroid, green – LC CRVT centroid, black – BMO centroid). Dashed lines represent the outline of the prelaminar CRVT (red), LC CRVT (green) and BMO margin (black). Blue double-sided arrows indicate the distance measured for the three relationships. (**C**) Example of the angle measurement (purple) for BMO-LC. X denotes the centroid for the BMO (black) and LC (green).

The BMO of each scan was delineated over the region of the ONH for each scan, and the center of the BMO was determined as the geometric center of the BMO. The 3-dimentional volume scans were separated into two portions at the level of the anterior LC surface (Fig. 1A). Projection images were produced from the prelaminar and LC sections, and the location of the vessel trunk at the level of the prelaminar and LC tissues were delineated manually, with the geometric center of the delineation serving the as the starting point from which the distance from the delineation was measured. If branching vessels existed at the level of prelaminar tissue, the distance between the center of the BMO and each of the individual vessels was averaged to provide one value for each eye. The following distances and angles (the direction relative to the centroid) were assessed (Fig. 1B): 1) the BMO centroid to the LC CRVT (BMO-LC), 2) the BMO centroid to the prelaminar CRVT (BMO-PL) and 3) the LC CRVT to the prelaminar CRVT (LC-PL). All angles were converted to that of the right eye (Fig. 1C).

### Statistical analysis

All statistical analyses was performed using R (version 3.2.3)^14^. A linear mixed effects model was used to assess differences in age, VF mean deviation (MD) and OCT RNFL thickness between diagnostic groups. A linear mixed effect model was also used to assess the effect of clinical diagnosis on the measured distances and angles while accounting for age and the use of both eyes in some subjects. A chi-square test was used to determine whether the distribution of the angles of 1) BMO-LC, 2) BMO-PL, and 3) LC-PL differed between diagnostic categories. P < 0.05 was considered statistically significant.

## Results

The characteristics of the study population are described in Table 1. There was a statistically significant difference in age, VF MD, and OCT mean RNFL thickness between the diagnostic groups (p < 0.01). There was no statistically significant association in the change of CRVT location in microns per MD increment for the three measured distances, BMO-LC, BMO-PL, and LC-PL. There was no statistically significant association in the change in microns per MD increment for the three measured distances, BMO-LC, BMO-PL, and LC-PL. The CRVT was identified at the prelaminar and LC level for all eyes. Examples of analyzed images from subjects with thin and thick prelaminar tissue and varying degrees of vessel distribution are provided in the supplementary figures.

**Table 1:** Study population characteristics.

	Healthy	Suspect	Glaucoma	P-value
Subjects/Eyes (n)	25/35	19/27	40/49	
Age (years)	39.3 ± 13.8	60.9 ± 7.6	66.0 ± 16.0	<0.01
VF MD (dB)	−0.17 ± 1.16	0.03 ± 0.80	−8.13 ± 7.30	<0.01
OCT RNFL (μm)	95.2 ± 15.4	89.2 ± 8.9	69.1 ± 11.0	<0.01

VF MD – visual field mean deviation; OCT RNFL – optical coherence tomography retinal nerve fiber layer.

**Figure 2:**
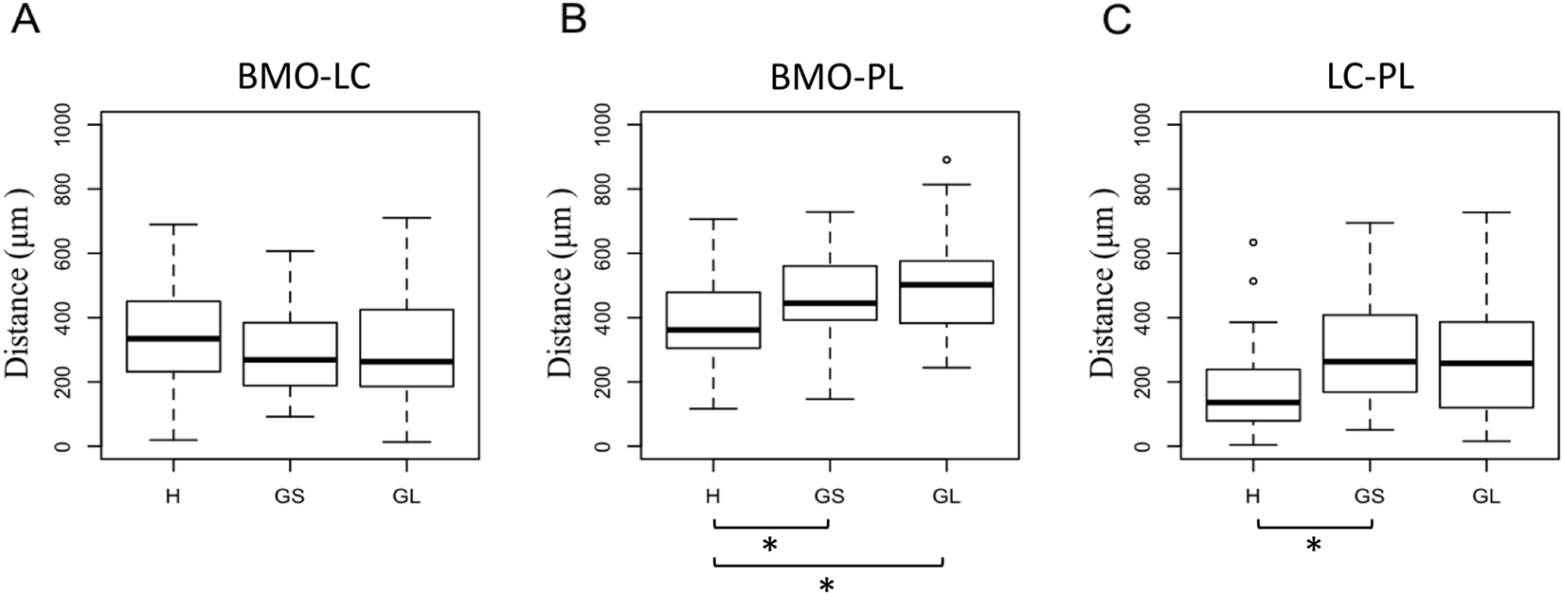
Boxplot for each diagnostic group showing the distance between (**A**) the center of the Bruch membrane opening (BMO) and the central retinal vessel trunk (CRVT) at the level of the lamina cribrosa (LC) (BMO-LC), (**B**) the center of the BMO and the CRVT at the level of the prelaminar tissue (BMO-PL), and (**C**) the CRVT at the level of the LC and the CRVT at the level of the prelaminar tissue (LC-PL). Asterisk denotes significant comparisons with p < 0.05.

No statistically significant difference was detected in BMO-LC among diagnostic groups (Fig. 2A). The BMO-PL of glaucoma and glaucoma suspect subjects were 153.1 ± 45.7 μm and 116.1 ± 45.7 μm greater (p = 0.001, 0.013, respectively) compared to healthy subjects (Fig. 2B). The LC-PL of glaucoma suspects was 118.7 ± 53.0 μm greater (p = 0.028) compared to healthy subjects, with no detectable difference between glaucomatous and healthy eyes (Fig. 2C).

**Figure 3:**
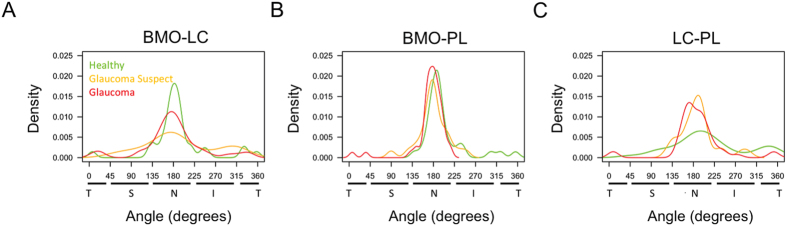
Distribution of (**A**) the angle between the central retinal vessel trunk (CRVT) at the level of the lamina cribrosa (LC) and center of the Bruch membrane opening (BMO-LC), (**B**) the angle between the CRVT at the level of the prelaminar tissue and center of the BMO (BMO-PL), and (**C**) the angle between the CRVT at the level of the LC and the CRVT at the level of the prelaminar tissue (LC-PL). Green denotes healthy, orange denoted glaucoma suspect, and red denotes glaucomatous eyes. 0° represents the horizontal temporal axis, with directionality proceeding clockwise through the superior, nasal, and inferior quadrants. Angle distribution was statistically significantly different between healthy eyes and both glaucoma suspect and glaucomatous eyes.

**Figure 4:**
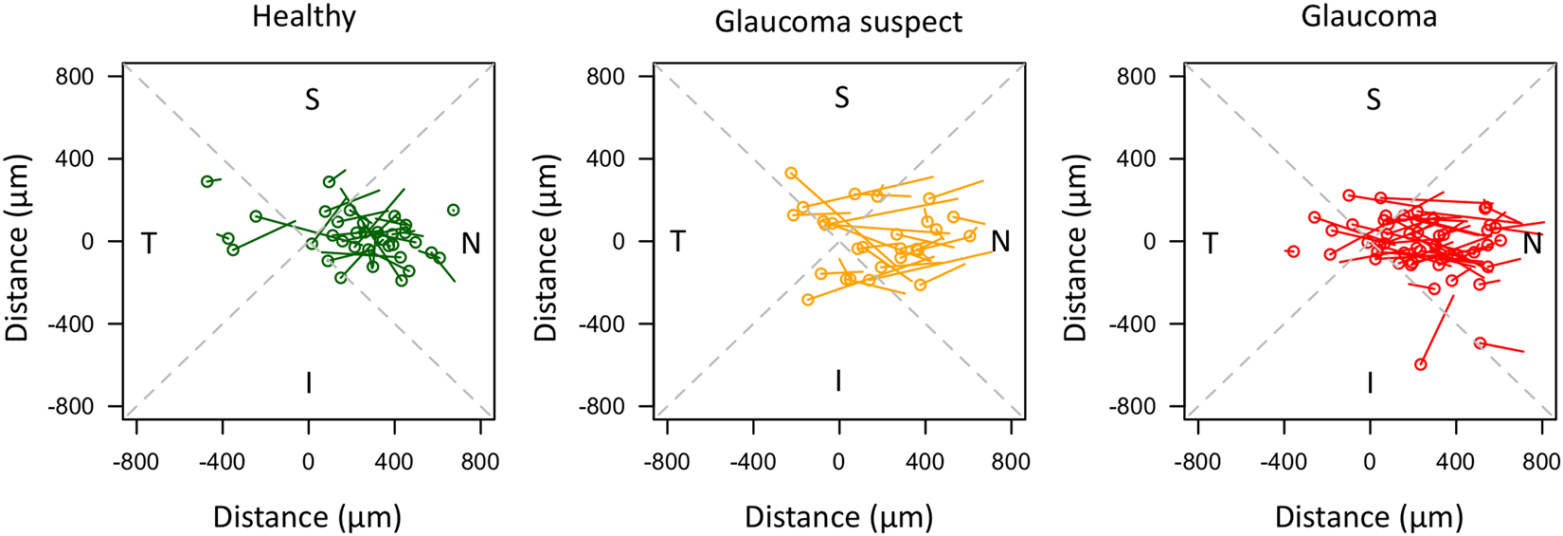
Directionality and magnitude of the path between the location of the central retinal vessel trunk (CRVT) at the level of the lamina cribrosa (LC) and the CRVT at the level of the prelaminar tissue for (**A**) healthy, (**B**) glaucoma suspect, and (**C**) glaucomatous eyes. The circle represents the CRVT location in the LC and the line ends at the CRVT location in the prelaminar tissue. The intersection of the gray dashed lines show the center of the Bruch membrane opening (BMO) and the dashed lines separate the plot into superior (S), inferior (I), temporal (T), and nasal (N) quadrants. All eyes have been converted to the orientation of the right eye.

The BMO-LC and BMO-PL angle distributions were not significantly different between diagnostic groups (Fig. 3A,B). The angle distribution of the LC-PL measurement was statistically significantly different between diagnostic groups, with glaucoma suspect and glaucoma subjects showing a more concentrated nasal location than healthy subjects (p < 0.01; Figs 3C and 4).

## Discussion

This study evaluated the *in-vivo* location of the CRVT within the LC and the pre-laminar tissue in healthy, glaucoma suspect, and glaucoma subjects. It was demonstrated that there was no difference in the location of the CRVT at the level of the LC between the diagnostic groups. However, glaucoma and glaucoma suspect subjects had vessels in the prelaminar region that were located significantly more nasally compared to healthy subjects.

As expected from previous clinical studies, the vast majority of blood vessels entered the LC near the center of the optic nerve, with a slightly nasal tendency (Fig. 4)^7^ Previous *ex vivo* ^15, 16^ and *in vivo* ^17^ studies have shown larger pores in the superior and inferior regions of the LC structure. The slightly nasal preference may be the result of the nasal side serving a relatively sparse population of axon bundles compared to the superior and inferior quadrants^18^.

The SS- OCT device used in this study offered significant advantages for the assessment of the CRVT compared to studies using fundus photographs. The 1060 nm light source of the SS-OCT improves penetration, reducing signal attenuation with depth^19^. While the vessel trunk entrance into the nerve can be seen with both OCT and fundus photography, a lack of true 3-dimentional imaging capability prevents the characterization of the trunk location at different ONH depths when using fundus photography alone. Looking forward, OCT angiography^20^ may present a useful tool capable of automatically assessing vessel abnormalities and changes at the level of the LC and prelaminar tissue.

A primary finding of this manuscript is that there is relatively little difference between the diagnostic groups with regards to how far the CRVT is from the center of the BMO at the level of the LC. While monkey studies demonstrate that the LC structure can undergo some remodeling^21, 22^, it is likely that it is not enough to appreciably affect the location of the CRVT. Loss of retinal ganglion cells results in a minimal shift in the CRVT at the level of the LC, despite marked cupping and nasal displacement of the vessels anterior to the LC.

Glaucomatous eyes exhibited a more nasal CVRT location at the level of prelaminar tissue. This nasal location can be seen as an increase in the distance of the CRVT from the center of the optic nerve (Fig. 2B). Furthermore, it is evident in Figs 3C and 4 that glaucoma and glaucoma suspect subjects have CRVT trajectories that mainly point nasally when plotted from the LC to prelaminar region, while healthy subjects had a much more varied trajectory. It is possible that the shift in retinal vessels seen in glaucoma is a result of the loss of retinal nerve fibers and thinning of the prelaminar tissue. This reduction in tissue could cause the vessels to be located more nasally and have a nasal orientation in relation to the nerve. Interestingly, the distance between the CRVT at the level of the LC and prelaminar tissue is significantly larger only for glaucoma suspects compared to healthy (Fig. 2C). It is possible that even though the location of the retinal vessels was not used as a criterion for a suspicious ONH, vessels shifts may have already been underway in these eyes, leading to these results.

As with other OCT assessments of the deep structures of the eye, we were limited by the depth of penetration currently possible with OCT technology. Therefore, in subjects without visible LC the location of the CRVT could not be ascertained, which may have create a bias as only subjects with visible LC were selected for analysis. While vessel shadowing can impede the ability to visualize deeper regions, especially in eyes with little change in vessel location between the prelaminar and laminar regions, it was still possible to identify the CRVT at the level of the LC since there was no movement in the vessel shadow.

As this is cross-sectional study, it is impossible to determine the time sequence for the change in the vessel location and the exact cause. While we suspect that the nasalization is due to loss of axons, it is possible that intraocular pressure pressing on the nerve could also have a direct effect on the vessels of the eye. However, this hypothesis is less likely because all subjects with glaucoma in this study were actively treated to lower their IOP to maintain it within a normal range. It is also possible that nasalization of vessels could be a cause, not a result of glaucomatous damage. Further longitudinal investigation is required to elucidate the answers to these questions.

In conclusion, we demonstrated *in vivo* that the shift of the retinal vessel in glaucoma suspect and glaucomatous eyes occurs at the level of the prelaminar tissue, with little effect on the CRVT position at the level of the LC. Improved characterization of vessel locations in glaucoma can improve our understanding of the disease process and its structural impact.

## Electronic supplementary material

Supplementary video legends

Supplemental Video 1

Supplemental Video 2
