# Supplementary material for: Location of the Central Retinal Vessel Trunk in the Laminar and Prelaminar Tissue of Healthy and Glaucomatous Eyes
Source: Sci Rep. 2017 Aug 30;7:9930. doi: 10.1038/s41598-017-10042-5 (PMC5577310; doi:10.1038/s41598-017-10042-5)
Supplement: Supplementary file 1 — Supplementary video legends [file 41598_2017_10042_MOESM1_ESM.pdf]

Location of the Central Retinal Vessel Trunk in the Laminar and Prelaminar Tissue of Healthy and Glaucomatous Eyes

Authors: Bo Wang[1,2*], Katie A Lucy[3*]; Joel S Schuman[3]; Hiroshi Ishikawa[3]; Richard A Bilonick[1,4]; Ian A Sigal[1,2]; Larry Kagemann[5]; Chen Lu[6]; James G Fujimoto[6]; Gadi Wollstein[3]

Affiliations: [1]UPMC Eye Center, Eye and Ear Institute, Ophthalmology and Visual Science Research Center, Department of Ophthalmology, University of Pittsburgh School of Medicine, Pittsburgh, PA;

[2]Department of Bioengineering, Swanson School of Engineering, University of Pittsburgh, Pittsburgh, PA;

[3]NYU Langone Eye Center, New York University School of Medicine, New York, NY;

[4]Department of Biostatistics, Graduate School of Public Health, University of Pittsburgh, Pittsburgh, PA;

[5]Center for Devices and Radiological Health, Food and Drug Administration, Silver Spring, MD;

[6]Department of Electrical Engineering and Computer Science, Massachusetts Institute of Technology, Cambridge, MA.

[*]These authors contributed equally to this work.

Supplemental Video 1

Example of CRVT tracing in a subject with thin prelaminar tissue. An OCT B-scan (left panel) shows the location (yellow line) of the C-mode (middle panel). The location of the CRVT is traced (right panel) with a yellow circle.

Supplemental Video 2

Example of CRVT tracing in a subject with thick prelaminar tissue. An OCT B-scan (left panel) shows the location (yellow line) of the C-mode (middle panel). The location of the CRVT is traced (right panel) with a yellow circle.
